# Applicability of crAssphage, pepper mild mottle virus, and tobacco mosaic virus as indicators of reduction of enteric viruses during wastewater treatment

**DOI:** 10.1038/s41598-020-60547-9

**Published:** 2020-02-27

**Authors:** Sarmila Tandukar, Samendra P. Sherchan, Eiji Haramoto

**Affiliations:** 1 Interdisciplinary Center for River Basin Environment, University of Yamanashi, 4-3-11 Takeda, Kofu, Yamanashi, 400-8511 Japan; 2 Department of Global Environmental Health Sciences, Tulane University, 1440 Canal Street, Suite 2100, New Orleans, LA 70112 USA

**Keywords:** PCR-based techniques, Water microbiology, Pathogens

## Abstract

This study was conducted to evaluate the applicability of crAssphage, pepper mild mottle virus (PMMoV), and tobacco mosaic virus (TMV) as indicators of the reduction of human enteric viruses during wastewater treatment. Thirty-nine samples were collected from three steps at a wastewater treatment plant (raw sewage, secondary-treated sewage, and final effluent) monthly for a 13-month period. In addition to the three indicator viruses, eight human enteric viruses [human adenoviruses, JC and BK polyomaviruses, Aichi virus 1 (AiV-1), enteroviruses, and noroviruses of genogroups I, II, and IV] were tested by quantitative PCR. Indicator viruses were consistently detected in the tested samples, except for a few final effluents for crAssphage and TMV. The mean concentrations of crAssphage were significantly higher than those of most tested viruses. The concentrations of crAssphage in raw sewage were positively correlated with the concentrations of all tested human enteric viruses (*p* <0.05), suggesting the applicability of crAssphage as a suitable indicator to estimate the concentrations of human enteric viruses in raw sewage. The reduction ratios of AiV-1 (1.8 ± 0.7 log_10_) were the lowest among the tested viruses, followed by TMV (2.0 ± 0.3 log_10_) and PMMoV (2.0 ± 0.4 log_10_). Our findings suggested that the use of not only AiV-1 and PMMoV but also TMV as indicators of reductions in viral levels can be applicable during wastewater treatment.

## Introduction

Wastewater treatment plants (WWTPs) are currently facing numerous issues regarding insufficient treatment of human enteric viruses during the treatment process^1^. Although a number of treatment procedures to remove physical, chemical, and microbiological waste from sewage are performed, these processess still need to be improved to reduce the viral content of water^2^. The advanced multiple-barrier concept has been proposed for wastewater reclamation and reuse; however, it is insufficient to achieve complete reduction of human enteric viruses^3,4^. In fact, advanced wastewater treatment has been developed to achieve reduction of viruses by facilitating chemical clarification, ultrafiltration, reverse osmosis, and advanced oxidation at different levels of regulatory control^5^. However, despite implementing these modifications, the efficiency with which treatment can reduce viral levels in wastewater is still unsatisfactory. Moreover, no authorized regulatory standard for the reduction of viruses in wastewater treatment has yet been established^6^.

Human enteric viruses are the leading cause of waterborne illnesses and usually transmitted via the fecal–oral route^5–7^. Infected individuals discharge millions of viral particles that ultimately enter sewage systems^7^. In this context, it is important to study the efficiency of virus reduction at WWTPs to ascertain whether all viruses are removed from effluent samples or not. Fecal indicator bacteria, such as total coliforms and *Escherichia coli*, are not reliable indicators of the presence and removal of human enteric viruses during wastewater treatment^8,9^. Human enteric viruses are resistant to adverse conditions, such as wide ranges of pH, low temperature, and chlorination^7^, making their removal difficult even using complex treatment technology including activated sludge process, oxidation ponds, activated carbon treatment, filtration and lime coagulation, and chlorination^10^.

Numerous studies have been carried out to establish a single indicator that can predict the presence of human enteric viruses in aquatic environments^7,11,12^. Previous studies successfully demonstrated that several types of viruses, such as human adenoviruses (HuAdVs), polyomaviruses, enteroviruses (EVs), Aichi virus 1 (AiV-1), and pepper mild mottle virus (PMMoV), can be used as potential indicators to illustrate the adequate reduction of viruses in wastewater^13–15^. Studies showed that PMMoV and tobacco mosaic virus (TMV) are the most abundant and frequently detected viruses in sewage^15–17^. They are distributed globally and more abundant in environmental samples than human entric viruses, without any pronounced seasonal variation^18,19^. It may thus be beneficial to use PMMoV to evaluate the performance of drinking water and wastewater treatment processes^15,20^, although no studies have been conducted to date to evaluate the efficiency of reduction of TMV during wastewater treatment. Given that AiV-1, a member of genus *Kobuvirus* in the family *Picornaviridae*^21^, is more abundant and reduced less than other viruses during the wastewater treatment process, it could be used as an indicator of virus reduction^15,22^. Similarly, based on studies conducted in Japan, among four individual genogroups (I–IV), F-specific RNA coliphages of genogroup I have been proposed as a possible indicator of reduction^23,24^. Selecting an appropriate viral indicator is still a major challenge due to various factors, such as human population dynamics, seasonal variations, and the types of treatment processes used^8^. Moreover, it is almost impossible to analyze all pathogenic viruses in wastewater at the same time due to lack of cost and time and laboring^25^.

CrAssphage, a bacteriophage infecting the human gut bacterium *Bacteroides intestinalis*^26^, was first discovered from human fecal microbiomes^27^. Human metagenomics has also demonstrated its abundance in the human gut^28^. Subsequently, quantitative PCR (qPCR) assays were developed and applied to quantify crAssphage in stool^29–31^ and environmental water samples^11,29,32–35^. Moreover, the sequences of crAssphage variants detected in Europe were found to differ from those in the USA^11^. Both assays revealed the abundance of crAssphage of human origin; however, lower quantities (~3 log_10_ lower) were also noted in samples of animal origin^11,29,33,36^, raising questions about the specificity of the assay. Although the appropriateness of crAssphage as a human fecal marker has been tested in many studies^11,29,32–35,37,38^, to the best of our knowledge, no studies evaluating crAssphage as an indicator of virus reduction during water treatment processes have yet been reported. Farkas *et al*.^37^ conducted one-year monitoring of crAssphage in influent and effluent of WWTPs in UK, reporting that crAssphage concentrations were 10^5^–10^9^ copies/L in influent and 10^7^–10^8^ copies/L in effluent. However, since the purpose of that study was also to evaluate the applicalibity of crAssphage as a human-derived wastewater contamination marker, no discussion is provided regarding the suitability of crAssphage as an indicator of virus reductions during wastewater treatment.

Based on this background, this study aimed to evaluate crAssphage along with PMMoV and TMV for their applicability as indicators of the reduction of human enteric viruses during wastewater treatment. In addition, the applicability of crAssphage to estimate the concentrations of human enteric viruses in wastewater samples was evaluated. To our knowledge, this is the first study to demonstrate the applicability of crAssphage and TMV as an indicator of virus reductions during wastewater treatment.

## Results

### Detection of viruses in three steps of WWTP

Seven of the eight human enteric viruses were detected in the tested samples at different frequencies, while noroviruses of genogroup IV (NoVs-GIV) were not detected in any of the examined samples, as shown in Table 1. Thirteen (100%) raw sewage, 11 (85%) secondary-treated sewage, and 12 (92%) final effluent samples were positive for at least one of the eight human enteric viruses tested. NoVs of genogroup I (NoVs-GI) and BK polyomaviruses (BKPyVs), which were present in 12 (92%) samples, were the most prevalent human enteric viruses in raw sewage. Six enteric viruses were detected in the secondary-treated sewage, where NoVs of genogroup II (NoVs-GII) (46%) were the most frequently detected enteric viruses. The concentrations of NoVs-GII were significantly higher than those of other human enteric viruses, such as EVs, NoVs-GI, and BKPyVs [analysis of variance (ANOVA); *p* < 0.05]. For the final effluent samples, NoVs-GI (7/13; 54%) were the most prevalent human enteric viruses.

**Table 1:** Detection of human enteric viruses and indicator viruses in wastewater samples.

Virus analyzed	No. of tested samples		Raw sewage		Secondary-treated sewage		Final effluent	
			No. of positive samples (%)	Concentration (mean ± standard deviation) (log_10_ copies/L)	No. of positive samples (%)	Concentration (mean ± standard deviation) (log_10_ copies/L)	No. of positive samples (%)	Concentration (mean ± standard deviation) (log_10_ copies/L)
Human enteric virus	AiV-1	13	9 (69)	5.0 ± 0.4	4 (31)	3.7 ± 0.3	5 (38)	3.8 ± 0.5
	EVs	13	11 (85)	4.8 ± 0.6	2 (15)	3.2 ± 0.2	1 (8)	3.4
	NoVs-GI	13	12 (92)	5.7 ± 0.5	5 (38)	4.2 ± 0.3	7 (54)	4.1 ± 0.4
	NoVs-GII	13	10 (77)	7.5 ± 0.8	6 (46)	6.1 ± 0.5	5 (38)	6.0 ± 0.2
	NoVs-GIV	13	0 (0)	Not applicable	0 (0)	Not applicable	0 (0)	Not applicable
	HuAdVs	13	11 (85)	6.7 ± 0.5	4 (31)	5.1 ± 0.3	5 (38)	5.2 ± 0.2
	JCPyVs	13	9 (69)	7.1 ± 0.7	0 (0)	Not applicable	3 (23)	6.0 ± 1.0
	BKPyVs	13	12 (92)	7.3 ± 0.6	3 (23)	5.4 ± 0.3	1 (8)	5.4
Indicator virus	CrAssphage	13	13 (100)	10.3 ± 1.3	13 (100)	7.7 ± 1.3	11 (85)	7.4 ± 1.1
	PMMoV	13	13 (100)	7.1 ± 0.5	13 (100)	5.4 ± 0.6	13 (100)	5.1 ± 0.7
	TMV	13	13 (100)	5.9 ± 0.5	13 (100)	4.3 ± 0.5	12 (92)	4.0 ± 0.5

Three indicator viruses (PMMoV, TMV, and crAssphage) were consistently detected in all wastewater samples tested, except for a few final effluents for TMV (38/39; 97%) and crAssphage (37/39; 95%). The mean concentrations of crAssphage in raw sewage, secondary-treated sewage, and final effluent were significantly higher than those of other tested viruses, except for BKPyVs and JC polyomaviruses (JCPyVs) in final effluent samples (ANOVA; *p* < 0.05).

### Reduction ratios of viruses during wastewater treatment process

**Figure 1:**
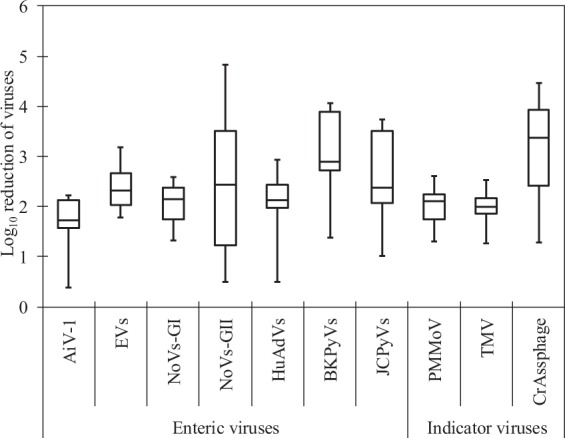
Annual reduction ratios of human enteric viruses and indicator viruses during the wastewater treatment process. Lines within the boxes represent median values, the upper and lower lines of the boxes represent 25th and 75th percentiles, respectively, and the bars outside the boxes represent minimum and maximum values.

Figure 1 shows the annual log_10_ reduction ratios of tested viruses (except for NoVs-GIV, which were not detected in any of the tested samples) during the whole wastewater treatment process. Among the seven human enteric viruses tested, BKPyVs showed the highest mean reduction ratio of 3.1 ± 0.8 log_10_ (n = 12), followed by NoVs-GII (2.5 ± 1.5 log_10_, n = 10), JCPyVs (2.5 ± 1.0 log_10_, n = 9), EVs (2.4 ± 0.5 log_10,_ n = 11), HuAdVs (2.1 ± 0.7 log_10_, n = 11), NoVs-GI (2.0 ± 0.4 log_10_, n = 12), and AiV-1 (1.8 ± 0.7 log_10_, n = 9) as the lowest. For indicator viruses, PMMoV (2.0 ± 0.4 log_10_, n = 13) and TMV (2.0 ± 0.3 log_10_, n = 13) showed comparable reduction ratios.

On the other hand, a higher reduction ratio was obtained for crAssphage (3.3 ± 1.0 log_10_, n = 13), which was the highest among all of the viruses tested in this study. Compared with this study, Farkas *et al*.^37^ reported lower reduction of crAssphage (1–2 log_10_) at two WWTPs tested (activated sludge and filter beds treatment), which suggests that the reduction efficiency of crAssphage during wastewater treatment can vary greatly depending on treatment processes. Further studies are needed to clarify the mechanism of reduction of crAssphage.

The mean reduction ratios of the tested viruses during primary–secondary treatment varied greatly from 1.7 ± 0.4 log_10_ (n = 13; for TMV) to 2.9 ± 0.8 log_10_ (n = 12; for BKPyVs), whereas those during chlorination varied from no reduction to maximum reduction of 0.7 ± 0.7 log_10_ (n = 13; for crAssphage).

The virus concentration values obtained from raw sewage were analyzed for any possible seasonality during the 13-month study period. For this purpose, the months were categorized into four seasons [i.e., summer (June–August), fall (September–November), winter (December–February), and spring (March–May)]. The concentrations of human enteric viruses and indicator viruses were relatively stable throughout the study period (Tukey–Kramer test; *p* > 0.05). High concentrations of NoVs-GII were observed in March, April, and December (8.4–8.6 log_10_ copies/L), but the results were not statistically significant (Tukey–Kramer test; *p* > 0.05). Total concentrations of human enteric viruses tested (7.7 ± 0.7 log_10_ copies/L) were also equally dissiminated in raw sewage, showing no apparent seasonal variation (Tukey–Kramer test; *p* > 0.05).

### Relationships between human enteric viruses and indicator viruses

To determine whether the concentrations of the indicator viruses were correlated with the concentrations of human enteric viruses tested, the data were analyzed for raw sewage. As shown in Table 2, a significant positive correlation was observed between the concentrations of human enteric viruses and those of each indicator virus in raw sewage, except between NoVs-GI and PMMoV and/or TMV.

**Table 2:** Relationship between the concentrations of human enteric viruses and of indicator viruses in raw sewage.

Virus analyzed	*r*-value		
	PMMoV	TMV	CrAssphage
AiV-1	0.66*	0.72*	0.88*
EVs	0.73*	0.75*	0.70*
NoVs-GI	0.30	0.46	0.70*
NoVs-GII	0.81*	0.85*	0.91*
HuAdVs	0.85*	0.72*	0.71*
JCPyVs	0.78*	0.87*	0.89*
BKPyVs	0.86*	0.88*	0.91*

^*^Statistically significant (*p* < 0.05).

## Discussion

Only a few studies have been conducted for the quantitation and reduction of crAssphage during wastewater treatment throughout the year^33,37^. CrAssphage, a recently identified human fecal marker, was detected in all wastewater samples tested, at significantly higher concentrations than human enteric viruses and other indicator viruses. Previous studies also reported that crAssphage was highly abundant in various environmental samples^11,29,33,34,36,39^. Despite its abundance with high concentrations in water samples, several studies reported cross-reactions with feces from different animals^11,29,34,38^, raising questions about its suitability as a human fecal marker.

The concentrations of EVs and AiV-1 in wastewater samples were lower than those of other human enteric viruses, in agreement with the results of previous studies conducted in New Zealand and the USA^40,41^. The ratio of positivity of AiV-1 (69%) in wastewater was comparable to those reported in previous studies^42,43^, which exhibited no seasonal variation^43^. AiV-1 has been detected from stools of gastroenteritis patients and wastewater in Japan and elsewhere^42–45^, suggesting its widespread presence in the environment. A consistently high prevalence of NoVs-GI and GII was noted from the wastewater samples tested, and their positivity ratios were comparable with those reported in previous studies^40,41,46–48^. This variation in the data may depend on the epidemiology of viruses or diseases in specific locations. However, unlike most previous studies^49–51^, no remarkable seasonal variation was found in the concentrations of NoVs in wastewater, although slightly higher concentrations were observed in March, April, and December. A previous study reported the observation of two peaks (October–December and March–May) in concentrations of NoVs-GII^52^, indicating that the seasonal trend can vary depending on the region. Concentrations of NoVs in the final effluent samples were found comparatively higher than those reported by Kitajima *et al*.^53^ and similar to those reported by Dias *et al*.^54^. The observation of higher concentrations and undefined seasonal variation of tested viruses in our study might be because of enrollment of the limited number of samples during the study period. Due to their high prevalence in sewage, HuAdVs and JCPyVs have been proposed as an indicator of fecal contamination. We found great variations in the concentrations and reduction values for these viruses compared with those of other tested indicator viruses (PMMoV, TMV, and crAssphage). The data were in agreement with previous studies conducted in Egypt^55^ and USA^53^. Molecular methods are rapid and reliable techniques for the detection and quantitation of viruses in environmental samples, but the presence of inhibitory substances in the sample itself could cause this variation during analysis^55^.

This study focused on a possibility of using crAssphage, along with PMMoV and TMV, as indicators of the reduction of human enteric viruses during wastewater treatment. For this purpose, the efficiency of reduction of the levels of these indicator viruses was determined and compared with that of human enteric viruses. In addition to PMMoV (2.0 ± 0.4 log_10_) and TMV (2.0 ± 0.3 log_10_), the reduction ratios of AiV-1 (1.8 ± 0.7 log_10_) were also found to be lower than those of other human enteric viruses tested. The variation in the reduction efficacy of viruses during operational conditions in the secondary treatment and chlorination depends on various factors, such as retention time, water temperature, and flow volume^56^. For example, secondary biological treatment processes typically achieve less than 2-log_10_ reduction of viruses^57^, while modified membrane bioreactor secondary treatment could achieve greater reductions (3.0 to >6.7 log_10_) than conventional secondary treatment (1.5–4.2 log_10_)^58^. One of the most important findings of this study is the demonstratation of the applicability of these viruses as indicators of human enteric virus reduction during the wastewater treatment process due to their lower reduction efficiencies. Previous studies proposed PMMoV and AiV-1 as viral indicators during the wastewater treatment process^15,21^, which agreed with the results of this study. Furthermore, this is the first study that quantified the occurrence of TMV at a WWTP, indicating the usefulness of this virus as an indicator.

Since this study was performed using only qPCR, another interesting issue is whether the viruses detected after chlorination remained infectious. Cell culture-based techniques are a gold standard to measure the infectivity of viruses, but suitable cultivation facilities are still not available for some viruses. Therefore, new and effective methods should be developed to evaluate the reduction of infectivity of non-cultivatable viruses^3^. Previous studies focused on the unsuccessful reduction of viruses by chlorination at WWTPs^15,41,49,50^.

In this study, the reduction ratio of crAssphage (3.3 ± 1.0 log_10_) was the highest among all tested viruses. Regardless of its remarkably high concentrations in wastewater and significant association with human enteric viruses, crAssphage cannot be proposed as an indicator of virus reduction during the wastewater treatment process due to its high reduction ratio. Similarly, BKPyVs, JCPyVs, and NoVs-GII were reduced more efficiently than other human enteric viruses, in agreement with the results of previous studies^15,22^. In some cases, human enteric viruses were not detected in secondary-treated sewage but were detected in final effluent, which was also reported in a previous study conducted in the USA^15^. Results of “no detection” are commonly obtained during the quantification of viruses at WWTPs^3,57^. This variation may occur due to the lack of homogeneity of viral particles or obstruction of the analysis by organic matter or suspended solids present in wastewater^57,58^. Moreover, the efficiency of virus removal also depends on the adsorptive affinities and the adsorbents such as sand, pure clays, bacterial cells, naturally occurring suspended colloids, estuarine silts, and sediments^59,60^.

In summary, this study successfully quantified all tested viruses in wastewater samples of a WWTP in the USA during a 13-month period. Three indicator viruses were consistently abundant year-round in the wastewater samples. Despite the abundance with high concentrations, crAssphage was judged to be inappropriate as an indicator of virus reduction because of its higher reductions than human enteric viruses. However, crAssphage was suggested to be a suitable indicator to estimate the concentrations of human enteric viruses in raw sewage. More studies need to be conducted to obtain a better understanding of the occurrence and reduction of crAssphage during the wastewater treatment process for the effective management of risks associated with wastewater in various geographical settings. Not only the previously proposed AiV-1 and PMMoV but also TMV were judged as appropriate indicators of the reduction of human enteric viruses. Further studies are needed to determine the reductions of crAssphage and TMV at WWTPs with different treatment systems.

## Materials and Methods

### Collection of wastewater samples

The WWTP in New Orleans, USA, treats 57,000,000 gallons of wastewater daily. A conventional treatment system combined with equipment for the activated sludge process is installed at this WWTP and disinfection was performed by chlorination. The final effluent is discharged from this WWTP into the Mississippi River, which is later filtered and used as a freshwater source for populations further down the river. Biochemical oxygen demand (BOD) values were 50–134 mg/L in the influent samples and <5 mg/L in the final effluent samples, pH of the final effluent was ~7.3. Raw sewage (before primary sedimentation), secondary-treated sewage (after sedimentation), and final effluent (after chlorination) were collected as grab samples monthly for a 13-month period between March 2017 and March 2018. Water samples (100 mL for raw sewage and 1,000 mL for secondary-treated sewage and final effluent) were collected in sterile bottles, transported to the laboratory in a cooler on ice, and processed within 6 h of sample collection.

### Virus concentration

Water samples were concentrated using an electronegative filter method, as previously described^61^ with slight modifications. Briefly, 2.5 M MgCl_2_ was added to these samples (100 mL of raw sewage and 1 L each of secondary-treated sewage and final effluent) to obtain a final concentration of 25 mM. Samples were subsequently passed through a mixed cellulose ester membrane filter (90-mm diameter and 0.45-µm pore size; Merck Millipore, Billerica, USA) attached to a glass filter holder (Advantec, Tokyo, Japan). Magnesium ions were removed by passing 200 mL of 0.5 mM H_2_SO_4_ through the filter, and the viruses were eluted with 10 mL of 1.0 mM NaOH. The eluate was recovered in a tube containing 50 µL of 100 mM H_2_SO_4_ and 100 µL of 100× Tris-EDTA buffer for neutralization. Further centrifugation was performed using a Centriprep YM-50 (Merck Millipore) to obtain a final volume of approximately 650 µL.

### DNA/RNA extraction and reverse transcription (RT)

Viral DNA and RNA were extracted using a ZR Viral DNA/RNA Kit (Zymo Research, Irvine, USA) to obtain a final volume of 100 µL, in accordance with the manufacturer’s protocol. RT was performed using a High Capacity cDNA Reverse Transcription Kit (Applied Biosystems, Foster City, USA).

### qPCR for viral genomes

In this study, HuAdVs, JCPyVs, BKPyVs, AiV-1, EVs, and NoVs-GI, GII, and GIV were tested as human enteric viruses, whereas crAssphage, PMMoV, and TMV were tested as indicator viruses. Briefly, 2.5 µL of the viral DNA or cDNA was mixed with 22.5 µL of a qPCR mixture containing 12.5 µL of Probe qPCR Mix (Takara Bio, Kusatsu, Japan), 0.4 µM each of forward and reverse primers, and 0.2 µM of a TaqMan (MGB) probe (Thermo Fisher Scientific, Waltham, USA). Subsequently, PCR tubes containing the mixtures were placed in a Thermal Cycler Dice Real Time System TP800 (Takara Bio) and incubated at 95 °C for 30 s, followed by 45 cycles of 95 °C for 5 s and 60 °C for 30 s for crAssphage^29^, HuAdVs^62^, BKPyVs, and JCPyVs^63^. Similarly, TaqMan (MGB)-based qPCR was performed for seven RNA viruses [AiV-1^64^, EVs^61,65^, NoVs-GI, GII^66^, GIV^67^, PMMoV^16,68^, and TMV^69^] with the following thermal conditions: 95 °C for 30 s, followed by 45 cycles of 95 °C for 5 s and 58 °C for 30 s (for AiV-1 and NoVs-GI and GII), 60 °C for 30 s (NoVs-GIV), or 60 °C for 60 s (for PMMoV, TMV, and EVs).

Six 10-fold dilutions of the artificially synthesized plasmid DNA were used to generate a standard curve, whereas molecular-grade water was used to prepare negative controls. Water samples, standard samples, and negative controls were tested in duplicate. The samples were considered negative if the cycle threshold value was greater than 40. One-tenth of the limit-of-detection value (2.0–2.4 log_10_ copies/L for secondary-treated sewage and final effluent) was given to a virus-negative sample. For calculation of the reduction ratios during primary–secondary treatment (from raw sewage to secondary-treated sewage), chlorination (from secondary-treated sewage to final effluent), and the whole treatment process (from raw sewage to final effluent), the results from months when a target virus was detected in the pretreated sample were used.

### Process control

As previously recommended^70^, coliphage MS2 (ATCC 15597-B1) was used as a molecular process control to evaluate the efficiency during extraction, RT, and qPCR. In brief, 1 µL of coliphage MS2 was inoculated to 100 µL of the virus concentrate, followed by DNA/RNA extraction and RT, as shown in the Section 2.3. Subsequently, a qPCR assay specific for F-specific RNA coliphages of genogroup I^71^, where MS2 belongs, was performed to quantify MS2-cDNA.

In addition, qPCR-control DNA was used to evaluate the level of inhibition during qPCR amplification. Artificially synthesized plasmid DNA containing sequences amplified by qPCR assays for chicken parvovirus^72^ was used for RNA viruses as a process control, as described previously^73,74^.

The efficiency was calculated from the ratio of the copy number of cDNA or plasmid DNA in the sample qPCR tube to that in the non-inhibition control tube. For MS2 molecular process control, the lowest extraction-RT-qPCR efficiency of 6.4% was observed for the secondary-treated sewage sample which had been collected in June 2017, and more than half of the tested samples (22/39, 56%) yielded the recovery of >100%. Similarly, the qPCR amplification efficiency ranged from 55% to 140%, with a mean of 86% (n = 39). These results indicate that there was no significant loss and/or inhibition during any of the detection procedures, such as DNA/RNA extraction, RT, and qPCR. However, there might have been any loss during virus concentration, which was not tested in this study.

### Statistical analysis

Data on the concentrations of all types of viuses was compared using ANOVA. Pearson’s correlation coefficients (*r*) were calculated to identify the relationships between total concentrations of human enteric viruses and concentrations of indicator viruses using bivariate correlation with Pearson’s coefficients. The Tukey–Kramer multiple comparison procedure was used to determine possible significant variation in the concentrations of indicator viruses and human enteric viruses among the seasons. A *p* value of <0.05 was considered significant.
